# The Application and Interpretation of IgG Avidity and IgA ELISA Tests to Characterize Zika Virus Infections

**DOI:** 10.3390/v11020179

**Published:** 2019-02-20

**Authors:** Fátima Amaro, María P. Sánchez-Seco, Ana Vázquez, Maria J. Alves, Líbia Zé-Zé, Maria T. Luz, Teodora Minguito, Jesús De La Fuente, Fernando De Ory

**Affiliations:** 1 European Programme for Public Health Microbiology Training (EUPHEM), European Centre for Disease Prevention and Control (ECDC), 17165 Solna, Sweden; fatima.f.amaro@gmail.com; 2 National Centre for Microbiology, Institute of Health Carlos III, 28220 Majadahonda, Spain; paz.sanchez@isciii.es (M.P.S.-S.); a.vazquez@isciii.es (A.V.); teoml@isciii.es (T.M.); jfuentel@isciii.es (J.D.L.F.); 3 Virored-Network for Emerging Viruses; m.joao.alves@insa.min-saude.pt; 4 Centro de Investigación Biomédica en Red de Epidemiología y Salud Pública (CIBERESP), 28029 Madrid, Spain; 5 National Institute of Health Doutor Ricardo Jorge, Centre for Vectors and Infectious Diseases Research 2965-575 Águas de Moura, Portugal; libia.zeze@insa.min-saude.pt (L.Z.-Z.); teresa.luz@insa.min-saude.pt (M.T.L.)

**Keywords:** Zika virus, dengue virus, secondary infections, cross-reactions, IgA, IgG avidity tests

## Abstract

In the absence of viremia, the diagnostics of Zika virus (ZIKV) infections must rely on serological techniques. In order to improve the serological diagnosis of ZIKV, ZIKV-IgA and ZIKV-IgG avidity assays were evaluated. Forty patients returning from ZIKV endemic areas, with confirmed or suspected ZIKV infections were studied. Samples were classified as early acute, acute and late acute according to the number of days post illness onset. Low avidity IgG was only detected at acute and late acute stages and IgA mostly at the early acute and acute stages. The date of sampling provides useful information and can help to choose the best technique to use at a determined moment in time and to interpret low avidity IgG and IgA results, improving the serological diagnosis of ZIKV.

## 1. Introduction

Zika virus (ZIKV), an emerging flavivirus transmitted mainly by mosquitos from the genus *Aedes*, is a cause of public health concern. Prior to 2015, ZIKV outbreaks occurred in Africa, Southeast Asia, and the Pacific Islands. In May 2015, the Pan American Health Organization issued an alert regarding the first confirmed ZIKV infections in Brazil [1]. Between 2015 and 2016, large outbreaks occurred in many countries in South and Central America as well as in the Caribbean [2]. Travelling for work or tourism between Portugal and Spain and Latin America is very common. Portugal reported ZIKV infections imported from Brazil for the first time in June 2015 [3]. In Spain, the first case of imported ZIKV infection was confirmed in January 2016 [4]. Shortly after, in March 2016, vertical transmission in an imported case was detected, in a 17-week pregnant woman [5]. 

ZIKV disease can be diagnosed within two weeks after onset of symptoms in serum or plasma, and up to three weeks in urine by real-time PCR (RT-PCR) [6]. However, in the absence of detectable ZIKV RNA or available molecular techniques, the only diagnostic methodology available is serology.

The current knowledge on ZIKV antibody kinetics is still limited. In addition, the cross-reactivity between ZIKV and other flaviviruses complicates serology. Thus, a positive result should always be confirmed by neutralization tests (NT), and even this assay is not always able to provide a definitive determination of the specific flavivirus causing the infection, particularly in individuals with a history of flavivirus infection or vaccination [6,7].

The co-circulation of DENV and ZIKV in some regions and the possibility of reinfections by the four DENV serotypes and/or ZIKV highlight the need for reliable serological tests when no molecular tools are available, or when viremia drops to undetectable levels in the patients. One way of discriminating infections between related viruses is to characterize IgG avidity [8]. Immunoglobulin G avidity is low after primary antigenic challenge but increases throughout progressively during subsequent weeks and months due to affinity maturation and antigen-driven B-cell selection. Avidity assays have been used to differentiate between acute or primary infection and persistent infection, recurrent infection, or reactivated disease in a number of infections and have been successfully used for flaviviruses such as West Nile and dengue viruses [8,9,10,11,12]. On the other hand, the IgA response has also been used as an indicator of recent and active infections in dengue and West Nile cases [13,14]. The objective of this study was to evaluate ZIKV IgG avidity and ZIKV IgA in their application of diagnosis of ZIKV infections.

## 2. Materials and Methods 

### 2.1. Studied Samples

A total of 79 serum samples, collected from 56 individuals were analyzed. These included 62 samples from 40 patients returning from ZIKV endemic areas in 2015–2016 and received for ZIKV diagnostics at the National Health institutes in Portugal and Spain. As controls, 16 patients (17 samples) with known recent or past DENV infection (15 collected in 2012, before the outbreak in the Americas, and one in 2016), were studied. The study was approved by the Ethical Committee of the Institute of Health Carlos III (code: CEI PI 70_2018).

These patients were organized in three groups: (I) 29 patients (43 samples) with ZIKV infection (cases confirmed by positive RT-PCR in at least one of the available samples for each patient) (Table 1); (II) 11 patients (19 samples) with serological suspicion of ZIKV infection (seroconversion and/or positive IgG and IgM and negative RT PCR) (Table 2); (III and IV) the control group, including patients with known recent (nine patients, 10 samples) or past (seven patients, seven samples) DENV infection (Table 3). 

Time elapsed after illness onset (days post symptom onset, dpso) was available for 60 samples (39 patients) in groups I and II, allowing us to classify the samples according to their infectious stage as early acute (≤5 dpso, *n* = 18), acute (6–20 dpso, *n* = 25) and late acute (>20 dpso, *n* = 17) [7,15].

### 2.2. Serological Diagnostic

IgM and IgG for DENV were determined using commercial ELISA (Panbio, Standard Diagnostics Inc., Geonggi-do, South Korea) (Spanish samples) or by in-house immunofluorescence as previously described [3] (Portuguese samples); IgM and IgG for ZIKV were determined using commercial ELISA kits (Euroimmun, Lübeck, Germany). DENV IgM in ELISA was confirmed by a background assay as described [10]. 

ZIKV-IgG avidity and ZIKV-IgA were tested by ELISA (Euroimmun, Lübeck, Germany) according to the manufacturer’s instructions. Samples with ratio avidity index (RAI) lower than 40% were regarded as having low avidity and those with RAI higher than 60% as having high avidity. Values between 40% and 60% were considered equivocal. Serum dilutions started at 1:101. As advised, when the OD was >1.2 without urea treatment and the RAI was >60%, we did fourfold dilutions, until the OD was <1.2, to avoid false high avidity results, according to manufacturer´s instructions. Whenever the OD reached a value of less than 0.140 without urea treatment after tentative dilutions, we considered as the final result the RAI’s of the last dilution with a valuable result. Results were deemed as inconclusive when IgG dropped to a negative value after the next dilution. For the ZIKV IgA assay samples were classified according to the ratio referred to a calibrator as positive (ratio was >1.1), equivocal (0.8–1.1) or negative (<0.8). All ZIKV ELISA tests were based on the NS1 protein.

Neutralizing antibodies were detected by an in house neutralization test, using Vero E6 cells and 100 TCID50% of ZIKV (strain MR-766). Samples were titrated in two-fold dilutions from 1/32. neutralization titers <1/32 were considered indicative of the absence of ZIKV neutralizing antibodies. 

Specificity and sensitivity were calculated for ZIKV-IgG avidity and ZIKV-IgA. These parameters were also calculated for both techniques together, considering as positive the patients positive for both approaches.

### 2.3. Molecular Diagnostics

RT PCR for ZIKV was performed with RealStar^®^Zika Virus (altona Diagnostics, Hamburg, Germany) at Institute of Health Carlos III (Spain) or with the RT-PCR as previously described [16] at the National Institute of Health Dr. Ricardo Jorge (Portugal). Positive results were confirmed in a second amplification using the method described by Balm and co-workers with slight modifications [17]. The performance in both countries has been evaluated through external quality assays [18] showing equivalent results.

## 3. Results

All patients in whom ZIKV infection was confirmed or suspected were infected in countries from the Caribbean, South and Central America except for patient P4 who had sexually acquired the infection (Table 1 and Table 2).

In group I (Table 1), regarding dpso, from 29 RT PCR positive samples, 15 were classified as early acute infections, 13 as acute and only one patient, a pregnant woman, presented a positive RT PCR result in a late acute infection (32 dpso). IgM was present in 14 samples (from 13 patients), ranging from 3–58 dpso (median 20), one at the early acute stage, six at acute stage and seven at the late acute stage. IgG was detected in 23 samples from 21 patients, ranging from 3–261 dpso (median 11), four at the early acute stage, 10 at the acute stage and nine at the late acute stage. Nine samples (from nine patients) presented low avidity (7–58 dpso, median = 21), three at acute and six at the late acute stage. Two patients presented high avidity results, at a late acute stage (36 and 261 dpso). IgA was present in 19 samples from 17 patients, ranging from 3–27 dpso (median 10), three at the early acute stage, 13 at acute stage and three at late acute stage. In this group, five samples from five patients presented DENV IgM positive results and 28 samples from 21 patients were positive for DENV IgG.

In group II (Table 2), there were 10 samples (from eight patients) with IgM positive results (9–45 dpso; median = 15). Eight of those samples were collected at acute stage, one at a late acute stage and one was unknown. Regarding IgG, 14 samples (10 patients) were positive (10–76 dpso; median = 24.5), six at acute stage, six at late acute stage and two unknown. Low avidity was observed in nine samples (8 patients; 10–49 dpso; median = 17.5) five at the acute stage and three at the late acute stage. A high avidity result was presented by a patient at 19 dpso (late acute stage). IgA was present in 11 samples from nine patients (7–19 dpso; median = 13), one at the early acute stage and eight at the acute stage. In Group II, three patients also showed DENV IgM and 15 samples (9 patients) presented DENV IgG.

In group III (Table 3), three patients showed ZIKV low avidity IgG (P44/S1, P45/S1 and P52/S1, and three were equivocal (P42/S1, P53/S1 and P56/S1). In relation to ZIKV IgA, only one sample (P56/S1) showed an equivocal result. All the other samples were negative for IgA.

**Figure 1:**
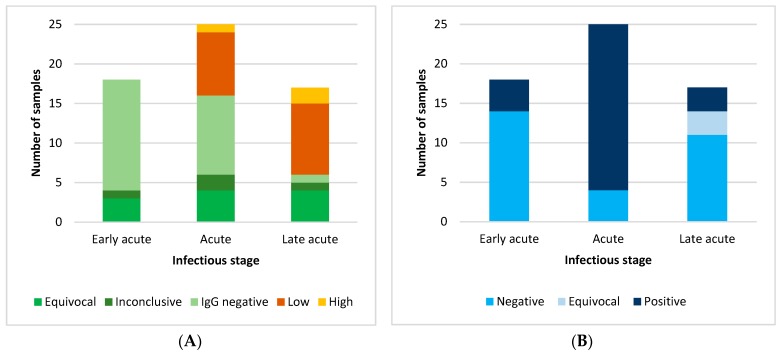
Serological testing in patients from groups I and II. (**A**) ZIKV IgG avidity results; (**B**) ZIKV IgA Results.

Figure 1A,B summarize the serological results for samples from groups I and II for low avidity IgG and IgA, respectively, related to the ZIKV infectious stages.

In their application to identify ZIKV recent infection cases, the avidity IgG testing showed a sensitivity value of 42.5% (17/40), (95% confidence interval [CI]: 28.5–57.8), being the specificity 62.5 (10/16) (95%CI: 38.6–81.5). Regarding ZIKV-IgA, sensitivity was 65% (26/40) (95%CI: 49.5–77.9) and specificity was 93.8% (15/16) (95%CI: 71.7–98.9). For both approaches simultaneously, the corresponding figures were 25% (10/40) (95% CI 14.2–40.2) and 100% (16/16) (CI95%: 80.6–100).

## 4. Discussion

All patients with suspected or confirmed ZIKV infection had traveled to countries with co-circulation of ZIKV and DENV. In our study five ZIKV PCR positive patients also presented with DENV-IgM positive results and/or low avidity and/or IgA positive results for ZIKV. Similar results were also obtained in Group II, with three patients presenting serological positive samples for both viruses. Steinhagen et al. [7], using the same assay we used here (NS1-based ELISA’s), found no cross-reactivity between ZIKV and DENV IgM. As such, our results can lead us to the suspicion of co-infection or, at least, shortly followed sequential infections with both viruses. Two cases of co-infection have already been reported in Pernambuco, Brazil, during a DENV outbreak in 2016 [19]. Other two in New Caledonia, 2014, one of those being autochthonous and the other a patient returning from the French Polynesia [20]. However, cross reactivity between ZIKV and DENV is well known [21,22]. In one study, sera from both DENV-naive and DENV pre-immune ZIKV patients strongly bound to ZIKV as well as DENV, with cross-reactive antibodies targeting both E and NS1 proteins. The four patient samples in that study were from primary ZIKV cases, where infection occurred during travel to ZIKV-afflicted areas [21]. Moreover, Lanciotti and colleagues [23] suggested that if ZIKV is causing secondary infections in a population with DENV (or other flaviviruses) background immunity, extensive cross-reactivity in the dengue IgM assay can occur. In this way, ultimate confirmation of co-infection would only rely on PCR positive results for both viruses. Unfortunately, by the time of this study, there was no sample available to perform DENV PCR. All these facts reinforce the need for serological tools to understand the time of infection in the absence of active viremia. This discrimination is very important due to the clinical role of ZIKV infections in pregnancy, which can result in fetal malformations, or in men that can transmit the virus sexually [24,25]. 

Overall, we had positive ZIKV-IgM results from day three to day 45. A recent similar study reported positive ZIKV-IgM results as early as day two and until 42 dpso [26]. Then again, Kadkhoda et al., using the same assay were able to detect positive IgM only at five dpso, testing a limited number of samples (*n* = 13) [27]. In the present study, ZIKV IgM was not detected in 13 out of 29 PCR positive patients. For seven of those 14 patients, the antibodies probably had not yet reached detectable levels, since samples were collected at the early acute stage (≤5 dpso), as previously reported [7] but we also cannot also exclude that the sensitivity of the technique may play its role in the results. Also, intrinsic immune factors cannot be discarded and might account for differences between individuals or populations, as reported by Lustig and colleagues who observed that ZIKV-IgM kinetics differed in Israeli, European and Chilean travelers [26]. Alternatively, the absence of IgM anti-ZIKV at acute stages concomitantly with high IgG ratio in some patients, may indicate a secondary infection. According to our cases, an IgG ratio > 2.0 was often associated with equivocal or inconclusive avidity IgG results and accompanied by a negative ZIKV-IgM result. Similar results were reported for DENV, where in secondary infections, the IgM response was variable or absent, accompanied by a dramatic increase in IgG antibodies [7,28]. Analogous kinetics has also been observed for ZIKV primary and secondary infections during the ZIKV epidemic in Micronesia in 2007 [23]. All these results strongly suggest than previous flavivirus infection could have an important impact on the interpretation of ZIKV avidity assay. According to the manufacturer of the kit, false high avidity values may be recorded due to high levels of antibodies, which may have been the case of patient P32, Table 2, whose follow up sample was taken 19 dpso. In contrast, the high avidity result for sample P27/S3, (Table 1), was expected since it represented a follow up sample taken nine months after onset of symptoms. 

In relation to IgA testing, positive results were obtained in nine samples (eight patients) from ZIKV infections, negative for IgM, two of them at the early acute stage, suggesting that IgA is detectable before IgM. Interestingly, Balmaseda and colleagues suggested that detection of IgA is more sensitive than IgM [29]. The majority of our ZIKV IgA positive results were obtained in samples collected at the early acute and acute stages and only three at the late acute stage (S21/S2, 27 dpso, P28/S2, 21 dpso and P29/S2, 24 dpso, Table 1). Individual kinetics may be observed but, in general, IgA seems to be of rapid appearance and short duration. As an example, in patient P33 with available paired samples (Table 2), there was a positive IgA result on day nine which turned negative on day 30. These results are in accordance with previous studies for other flaviviruses [13,14,27]. These facts can indicate that ZIKV IgA might support recent ZIKV infection, thereby adding a tool for acute ZIKV infection diagnostics with acceptable specificity and high sensitivity, as suggested by Zhang and colleagues [30]. 

Nearly all the patients in Group II showed some indicators of recent ZIKV infection even though samples had not been tested or were PCR-negative (Table 2). Thus, all the patients in this group could be considered infected by ZIKV.

In groups III and IV (recent and past DENV infections) three patients showed ZIKV low avidity IgG (samples P44/S1, P45/S1 and P52/S1, Table 3). One of them, P44, worked on a cruise ship and was exposed to DENV, acquiring the infection during the outbreak occurred in Madeira Island [31]; this patient could have travelled to various countries and been exposed to other flaviviruses, namely by vaccination for Yellow Fever, Japanese Encephalitis or Tick-Borne Encephalitis viruses. For the other two patients there was no available information and thus we cannot totally exclude the possibility of a ZIKV infection in none of them, in despite of the year in which the samples were taken (before the Zika outbreak in the Americas). The three low and three equivocal ZIKA avidity results from Table 3, that are from patients assumed to have solely a dengue or other flavivirus infection, illustrates the on-going cross-reactivity within the flavivirus group. No positive results were obtained using the IgA assay (only a sample showed an equivocal one), thus ensuring the specificity of this approach. 

The main limitation of this paper was the selection of samples, especially the controls. Control cases were retrospectively selected as having a recent or past DENV infection, prior to the ZIKV epidemics in the Americas. This selection was based on the fact that both viruses share epidemiological and clinical issues, and a high degree of cross reactivity. Furthermore, the selection of the controls did not consider the time elapsed after the onset, making difficult the comparison of cases and controls. The selection of samples has, as well, impact on the sensitivity and specificity of the assays in evaluation, being especially important for the calculation of specificity. 

## 5. Conclusions

Simultaneous ZIKV IgM, low avidity IgG and IgA positive results seem to corroborate a recent ZIKV infection. However, the serological diagnosis of ZIKV in symptomatic patients should be supported by careful inquiry at the time of admission. The date of symptom onset together with the date of sample collection thus provides useful information and may help interpret ZIKV low avidity IgG and IgA. In the early acute and acute stages of the infection, IgA tests seem to be more valuable, while low avidity IgG appears to be more useful at the acute and late acute stages. Lastly, the combined use of both techniques seems preferable in order to avoid false positive results. Further studies, with a larger number of patients, with follow up samples, would be necessary to clarify the kinetics of ZIKV antibodies, and the maturation of IgG avidity. 

## Figures and Tables

**Table 1:** Group I-PCR and serological results for patients with ZIKV infection.

Group	ID/Sample Number	Country of Infection	DPSO	PRNT (ZIKV)	ZIKV RT-PCR	ZIKV IgM Ratio (ELISA)	ZIKV IgG Ratio (ELISA)	ZIKV RAI (Last Dilution)	IgA Ratio (ELISA)	DENV IgM *	DENV IgG *
I	P1/S1	Venezuela	32	ND	Pos (u)	Neg	Neg		Neg	ND	Neg
I	P1/S2		58	ND	ND	Pos (2.0)	Pos (3.9)	L (35.9)	Equiv (1.0)	ND	Pos
I	P2/S1	Honduras	7	ND	Pos (s)	Neg	Neg		Neg	Neg	Neg
I	P2/S2		21	Pos	ND	Pos (2.5)	Pos (3.9)	L (33.4)	Neg	ND	Pos
I	P3/S1	Dominican Republic	3	Pos	Pos (u)	Neg	Neg		Neg	Neg	Neg
I	P3/S2		12	Pos	Neg (s)	Pos (3.5)	Pos (1.3)	L (18.2)	Pos (2.6)	Neg	Neg
I	P4/S1	Sexual transmission	0	ND	Pos (u)	Neg	Neg		Neg	ND	Pos
I	P4/S2		12	ND	Neg (s)	Pos (1.4)	Pos (2.9)	L (15.6)	Pos (5.3)	ND	Pos
I	P5/S1	Brazil	0	ND	Pos (u)	Neg	Neg		Neg	Neg	Pos
I	P5/S2		32	ND	ND	Pos (1.7)	Pos (2.8)	L (38.6)	Neg	ND	Pos
I	P6/S1	Unk	4	ND	Pos (b,u)	Neg	Neg		Neg	Neg *	Pos *
I	P6/S2		26	ND	ND	Pos (1.2)	Pos (3.2)	L (38.8)	Neg	Neg *	Pos *
I	P7/S1	Brazil	4	ND	Pos (b)	Neg	Neg		Neg	Neg *	Pos *
I	P7/S2		7	ND	ND	Neg	Pos (6.9)	Equiv (53.6)	Pos (4.2)	Neg *	Pos *
I	P8/S1	Honduras	4	ND	Pos (u)	Neg	Pos (3.3)	Equiv (40.6)	Neg	Neg	Pos
I	P9/S1	Honduras	3	Pos	Pos (u)	Neg	Pos (6.4)	Inc (72.6)	Pos (2.7)	ND	Pos
I	P10/S1	Venezuela	11	Pos	Pos (u)	Neg	Pos (4.6)	Equiv (41.3)	Pos (2)	Neg	Pos
I	P11/S1	Mexico	1	ND	Pos (u)	Neg	Neg		Neg	ND	Neg
I	P12/S1	Colombia	7	Pos	Pos (u)	Pos (6.4)	Pos (2.7)	L (24.3)	Pos (2.9)	Neg	Neg
I	P13/S1	Colombia	3	ND	Pos (u)	Pos (2.4)	Pos (2.2)	Equiv (54.6)	Pos (1.2)	ND	Pos
I	P14/S1	Martinica	7	ND	Pos (u)	Neg	Neg		Neg	Neg *	Pos *
I	P14/S2		21	ND	ND	Pos	Equiv (1.0)	Inc (71.3)	Neg	Neg *	Pos *
I	P15/S1	Brazil	12	ND	Pos (u)	Neg	Pos (6.2)	Inc (73.7)	Pos (4.6)	Neg *	Pos *
I	P16/S1	Brazil	20	ND	Pos (u)	Neg	Pos (5.9)	Inc (64.6)	Pos (1.7)	Neg *	Pos *
I	P17/S1	Brazil	3	ND	Pos (u)	Neg	Neg		Neg	Neg *	Neg *
I	P18/S1	Brazil	5	ND	Pos (b,u)	Neg	Neg		Neg	Neg *	Neg *
I	P19/S1	Unk	19	ND	Pos (u)	Pos (1.4)	Neg		Pos (1.2)	Neg *	Neg *
I	P20/S1	Brazil	3	ND	Pos (u)	Neg	Neg		Neg	Neg *	Pos *
I	P21/S1	Bolivia	6	ND	Pos (u)	Neg	Pos (5.1)	Equiv (51.0)	Pos (4.7)	Neg	Pos
I	P21/S2		27	Pos	ND	Neg	Pos (5.7)	Equiv (57.8)	Pos (1.2)	Neg	Pos
I	P22/S1	Dominican Republic	7	ND	Pos (u)	Neg	Pos (7.1)	Equiv (53.9)	Pos (2.6)	Neg	Pos
I	P22/S2		36	ND	ND	Neg	Pos (6.2)	H (80.4)	Neg	ND	Pos
I	P23/S1	unk	9	ND	Pos (u)	Neg	Neg		Neg	Neg *	Neg *
I	P24/S1	unk	10	ND	Pos (u)	Neg	Neg		Neg	Neg *	Pos *
I	P25/S1	Colombia	<20	ND	Pos (s)	Neg	Pos (2.8)	Equiv (59)	Pos (1.2)	Pos	Pos
I	P26/S1	Venezuela	4	ND	Pos (u)	Neg	Pos (5.0)	Equiv (43.5)	Pos (3.4)	Pos	Pos
I	P27/S1	Brazil	3	ND	Pos (u)	Neg	Neg		Neg	Neg *	Neg *
I	P27/S2	Brazil	9	ND	ND	Pos (4.6)	Neg		Pos (2.6)	Pos *	Neg *
I	P27/S3		261	ND	ND	Neg	Pos (1.4)	H (72.8)	Neg	Neg *	Pos *
I	P28/S1	Brazil	5	ND	Pos (u)	Neg	Neg		Neg	Neg *	Neg *
I	P28/S2		21	ND	ND	Pos (3.5)	Pos (1.7)	L (29.4)	Pos (4.4)	Pos *	Pos *
I	P29/S1	Colombia	6	ND	Pos (u)	Pos (2.6)	Equiv (1.0)	Equiv (54.8)	Pos (4.8)	Neg *	Pos *
I	P29/S2		24	ND	ND	Pos (2.0)	Pos (1.5)	L (33.4)	Pos (4.3)	Pos *	Pos *

DPSO—Days post illness onset; Equiv—Equivocal; Inc—inconclusive; L—low avidity; ND—not determined; Neg—negative result; Pos—positive result; PRNT—plaque reduction neutralization test; RAI—Relative avidity index; unk—unknown; (b)—blood sample; (s)—serum sample; (u)—urine sample. DENV IgM and IgG: the samples marked * were tested by IIF, the remaining by ELISA.

**Table 2:** Group II. Patients with serological suspicion of ZIKV infection.

Group	ID/Sample Number	Country of Infection	DPSO	PRNT (ZIKV)	ZIKV RT-PCR	ZIKV IgM Ratio (ELISA)	ZIKV IgG Ratio (ELISA)	ZIKV RAI (Last Dilution)	IgA Ratio (ELISA)	DENV IgM *	DENV IgG *
II	P30/S1	Dominican Republic	4	ND	ND	Neg	Neg		Neg	Neg	Neg
II	P30/S2		32	ND	ND	Neg	Pos (2.9)	L (30.4)	Neg	Neg	Neg
II	P31/S1	Brazil	≤7	ND	ND	Equiv (1.1)	Neg		Pos (1.2)	Neg	Pos
II	P31/S2		≤18	Pos	ND	Pos (4.6)	Pos (3.1)	L (16.1)	Pos (5.1)	ND	Pos
II	P32/S1	Venezuela	2	ND	ND	Neg	Neg		Neg	Neg	Pos
II	P32/S2		19	Pos	ND	Pos (1.3)	Pos (6.1)	H (65.8)	Pos (5.0)	Neg	Pos
II	P33/S1	Unk	9	ND	Neg (b)	Pos (2.57)	Neg		Pos (4.1)	Neg *	Neg *
II	P33/S2		30	ND	ND	Equiv (1.0)	Pos (2.9)	L (32.4)	Neg	Neg *	Neg *
II	P34/S1	Venezuela	49	Pos	ND	Neg	Pos (2.9)	L (30.2)	Neg	Pos	Pos
II	P34/S2		76	Pos	ND	Neg	Pos (3.6)	Equiv (44.9)	Neg	Neg	Pos
II	P35/S1	Colombia	unk	Pos	Neg (s)	Pos (7.4)	Pos (3.7)	L (23.4)	Pos (4.1)	Pos	Pos
II	P36/S1	Brazil	10	ND	Neg (b,u)	Pos (2.5)	Pos (1.4)	L (31.6)	Pos (5.2)	Pos	Pos
II	P37/S1	Brazil	13	ND	Neg (b)	Pos (4.2)	Neg		Pos (1.3)	Neg *	Pos *
II	P38/S1	Brazil	13	ND	Neg (b)	Pos (2.8)	Pos (1.9)	L (18.2)	Pos (5.1)	Neg *	Pos *
II	P38/S2		15	ND	ND	Pos (1.6)	Pos (3.0)	L (29.4)	Pos (2.47)	Neg *	Pos *
II	P39/S1	Dominican Republic	17	ND	Neg (s)	Pos (2.4)	Pos (3.8)	L (38.1)	Pos (4.55)	ND	Pos *
II	P39/S2		45	Pos	Neg	Pos (2.5)	Pos (2.8)	Equiv (45.5)	Equiv (0.97)	ND	Pos
II	P40/S1	Dominican Republic	unk	ND	ND	Equiv (1.0)	Pos (6.4)	Inc (72.5)	Pos (1.96)	ND	Pos
II	P40/S2		67¨	ND	Neg (s)	Neg	Pos (6.1)	H (68.6)	Neg	ND	Pos

DPSO—Days post illness onset; Equiv—Equivocal; Inc—inconclusive; L—low avidity; ND—not determined; Neg—negative result; Pos—positive result; PRNT—plaque reduction neutralization test; RAI—Relative avidity index; unk—unknown. (b)—blood sample; (s)—serum sample; (u)—urine sample. DENV IgM and IgG: the samples marked * were tested by IIF, the remaining by ELISA.

**Table 3:** PCR and serological results for patients with recent DENV infections (Group III) and with past DENV infections (Group IV).

Group	ID/Sample Number	DPSO	Country of Infection	PRNT (ZIKV)	ZIKV RT-PCR	ZIKV IgM Ratio (ELISA)	ZIKV IgG Ratio (ELISA)	ZIKV RAI (Last Dilution) (ELISA)	IgA Ratio (ELISA)	DENV IgM (ELISA)	DENV IgG (ELISA)
III	P41/S1	Unk	Unk	ND	ND	Neg	Neg		Neg	Pos	Neg
III	P42/S1	Unk	Unk	ND	ND	Neg	Pos (1.5)	Equiv (45.2)	Neg	Pos	Pos
III	P43/S1	Unk	Cambodia	ND	ND	Neg	Neg		Neg	Pos	Pos
III	P44/S1	Unk	Cruise with a stop at Madeira Island	ND	ND	Neg	Pos (2.9)	L (27.4)	Neg	Pos	Pos
III	P45/S1	Unk	Americas	ND	ND	Neg	Pos (1.3)	L (10.4)	Neg	Pos	Pos
III	P46/S1	Unk	Unk	ND	ND	Neg	Neg		Neg	Pos	Pos
III	P47/S1	Unk	Dominican Republic	ND	ND	Neg	Neg		Neg	Pos	Pos
III	P48/S1	Unk	Unk	ND	ND	Neg	Neg		Neg	Pos	Pos
III	P49/S1	8	Unk	ND	ND	Neg	Neg		Neg	ND	Pos
III	P49/S2	13	Unk	ND	ND	Neg	Neg		Neg	Pos	Pos
IV	P50/S1	Unk	Cambodia	ND	ND	Neg	Equiv (0.9)		Neg	Neg	Pos
IV	P51/S1	Unk	Unk	ND	ND	Neg	Neg		Neg	Neg	Pos
IV	P52/S1	Unk	Unk	ND	ND	Neg	Pos (1.5)	L (39.4)	Neg	Neg	Pos
IV	P53/S1	Unk	Unk	ND	ND	Neg	Pos (1.2)	Equiv (41.8)	Neg	Neg	Pos
IV	P54/S1	Unk	Venezuela	ND	ND	Neg	Neg		Neg	Neg	Pos
IV	P55/S1	Unk	Unk	ND	ND	Neg	Neg		Neg	Neg	Pos
IV	P56/S1	Unk	Unk	ND	ND	Neg	Pos (1.4)	Equiv (41.2)	Equiv (0.96)	Neg	Pos

DPSO—Days post illness onset; Equiv—Equivocal; Inc—inconclusive; L—low avidity; ND—not determined; Neg—negative result; Pos—positive result; PRNT—plaque reduction neutralization test; RAI—Relative avidity index; unk—unknown.
